# Steam Generator Maintenance Robot Design and Obstacle Avoidance Path Planning

**DOI:** 10.3390/s25020514

**Published:** 2025-01-17

**Authors:** Fengwei Yuan, Gengzhen Ren, Qian Deng, Xiangjiang Wang

**Affiliations:** 1 College of Resource Environmental and Safety Engineering, University of South China, Hengyang 421001, China; fengweiyuan@usc.edu.cn; 2 College of Mechanical Engineering, University of South China, Hengyang 421001, China; 20222006110355@stu.usc.edu.cn (G.R.); dengqian@usc.edu.cn (Q.D.); 3 Hunan Provincial Key Laboratory of Emergency Safety Technology and Equipment for Nuclear Facilities, University of South China, Hengyang 421001, China

**Keywords:** steam generator, ten degrees of freedom, plate plugging robot, forward and inverse kinematics, path planning

## Abstract

To solve the issue of inconvenient and dangerous manual operation during the installation and removal of the main pipe plugging plate in the steam generator in nuclear power plants, a ten-degree-of-freedom plugging robot was designed in the present study that includes a collaborative robotic arm coupled with a servo electric cylinder. By establishing a joint coordinate system for the robot model, a D-H parameter model for the plate plugging robot was established, and the forward and inverse kinematics were solved. The volume level approximate convex decomposition algorithm was used to fit the steam generator model with a convex packet, and an experimental simulation platform was constructed. Lastly, path planning was carried out by using the RRT algorithm, with the paths divided into three phases for analysis. The simulation results show that the path in the first stage is relatively smooth, and the parameter changes in each joint are relatively stable. The path in the second stage exhibits zigzagging, with the parameter change curve of joint 9 being particularly evident, and the path in the third stage still exhibits zigzagging—the parameter change curves of joints 3 and 10 are particularly evident. The results of the present study show that, although the paths show a certain degree of zigzagging in complex environments, the plate plugging robot is still able to automatically complete the plate plugging task while avoiding obstacles, which greatly reduces the risk posed to the operator when exposed to a high-radiation environment, in addition to having certain research and application value.

## 1. Introduction

In order to ensure the safe operation of nuclear power plant reactors and enhance emergency disaster relief capabilities, the application of robots in nuclear power plants has become a priority in numerous countries. One of the key pieces of equipment in nuclear power plants is the steam generator, which requires regular maintenance to ensure nuclear safety [1,2,3]. Westinghouse, a company in the United States, developed the Pegasys robot [4], and due to its special structural design, this robot is widely used to repair faults in steam generators. Researchers at Japan’s Waseda University developed a robot that can be used to treat radioactive wastewater and adapted it to some of the valves on a pipeline [5]. French scientists developed a special robot, named Roger, that is specially used for the daily maintenance and repair of steam generators, and, simultaneously, they were able to solve some difficulties in the remote control of the robot [6]. Wu et al. designed a set of six-degree-of-freedom steam generator maintenance manipulators to meet the auxiliary maintenance needs of water chambers [7]. The removal of steam generator plugging plates is also one of the necessary operations for the shutdown and maintenance of nuclear power plants. In current maintenance operations, the sealing operation of a steam generator’s low-water plugging plate requires workers to bring the collapsible plugging plate into the interior of the steam generator and cover it on the corresponding plugging port; thereafter, maintenance staff can access the plugging plate to carry out subsequent maintenance operations. However, such operations take a long time to perform in a high-intensity radiation environment [8]. Based on development trends in the nuclear power industry, in the future, robots will replace the manual installation of plugging plates in order to improve operational efficiency and ensure the safety of staff. For example, the ADAM robot developed in South Korea adopts a stable hydraulic transmission method and can complete the installation of the main pipeline plugging plates in a high-radiation environment [9]. The seven-degree-of-freedom evaporator overhaul robot developed by the Nuclear Power Research Institute of Harbin Engineering University consists of a propulsion device and a six-degree-of-freedom industrial robotic arm, which can complete the entire process of encapsulating the end effector into the main pipeline hole in the water chamber [10]. The robot developed by the Beijing University of Technology was realized by modifying the plugging plate. A lightweight robot was installed on the plugging plate for the installation and removal of the plugging plate bolts [11]. Based on the analysis of the working conditions of plugging plate removal, we propose a robot program that can enter the interior of the water chamber through a narrow manhole, which consists of seven rotating joints and three moving joints to form a ten-degree-of-freedom robotic structure that is able to move flexibly in special environments.

For the control of maintenance robots, due to the narrow space inside steam generators, most companies still use a demonstration-type robotic arm. In path planning, it is necessary to demonstrate the operation of the demonstrator before starting any work and record the relevant work parameters before the robot can achieve automation [12]. To achieve this, it is necessary for workers to employ the demonstrator first in the nuclear radiation environment. This way of working can cause significant physical harm to the workers involved; therefore, it is important to automate the entire workflow of the plugging robot. To solve the issue of automatic path planning in robots, numerous scholars have conducted related research and proposed path planning methods such as the artificial potential field method [13,14], A* algorithm [15,16], particle swarm algorithm [17], genetic algorithm [18], RRT [19,20,21,22], and PRM [23,24,25]. Xu et al. proposed a motion planning method for a quadruped maintenance robot for the inspection of a steam generator’s heat exchange tubes. The base trajectory was planned through kinematic solution search, the foothold motion evaluation system was established through combination with a similar preference ranking technique, and the path was segmented [26]. Li et al. proposed an adaptive stepping RRT* (AS-RRT*) algorithm for improving the efficiency of a tea-picking robotic arm’s path planning and obstacle avoidance [27]. Xia et al. proposed an improved velocity potential field (IVPF) algorithm to address safety and efficiency issues in trajectory planning for a medical–surgical collaborative robot, with an improved velocity potential field algorithm being proposed. By introducing direction factors, obstacle velocity factors, and tangential velocity, the IVPF algorithm effectively avoids the local minimum problem of the traditional velocity potential field algorithm and enhances dynamic obstacle avoidance capabilities [28]. In this study, we optimized the plugging plate installation process. The RRT algorithm performs path planning on a simulation platform. If the work path can be followed, the relevant results can be used directly during the work process, with it no longer being necessary for workers to manually enter the radiation zone. Undoubtedly, this brings about significant improvements for the worker’s working environment, making it possible for them to disassemble and install the plugging plate in the steam generator in a more secure and stable way. Therefore, this study has certain research and application value.

The present paper is formatted as follows: in Section 1, the overall structure of the designed plate plugging robot is introduced, the structural parameters of its components are described, a kinematic analysis is performed, and lastly, the relevant mathematical model for analysis and discussion is established; in Section 2, the process of building the simulation environment is described and the volume hierarchy approximate convex decomposition algorithm is applied in the convex packet fitting for obstacles, which simplifies the model’s complexity; in Section 3, the RRT algorithm is used and the path planning simulation of the plate plugging robot is performed so that it can automatically avoid obstacles to complete the plate plugging installation task, and its motion trajectory and the change curve of each joint parameter are obtained; and lastly, in Section 4, the results of the simulation experiments are discussed.

## 2. Kinematic Analysis of the Plate Plugging Robot

### 2.1. Robot and Steam Generator Modeling

**Figure 1:**
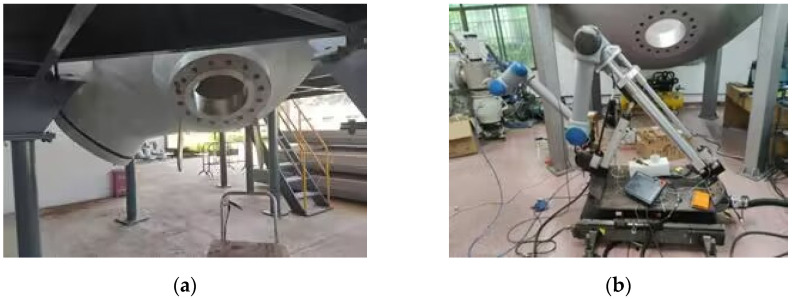
Experimental setup: (**a**) steam generator and (**b**) plate plugging robot.

**Figure 2:**
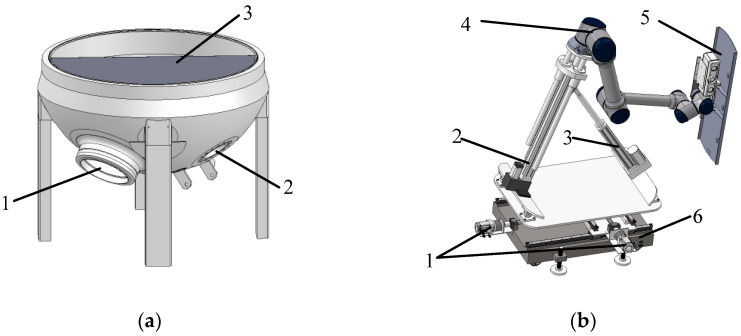
Model of the experimental setup: (**a**) steam generator model: 1. plug hole; 2. manhole; 3. bulkhead; (**b**) plate plugging robot model: 1. screw module; 2. feed electric cylinder; 3. angle adjustment electric cylinder; 4. collaborative robotic arm; 5. plugging plate; 6. moving platform.

The physical and modeled steam generator and plate plugging robot are shown in Figure 1a and Figure 2a. The robot needs to feed the plate plugging robot through the manhole, and after a series of motion transformations, it covers the plugging plate at the specified position of the plug opening to complete the task. Due to the fact that the diameter of the manhole of the steam generator is only 400 mm and the plugging port is roughly 1000 mm in size, the required plugging plate will be larger, and due to the narrow space inside the generator, the motion path of the robot will need to be analyzed and studied in order to determine the optimal working path.

**Figure 3:**
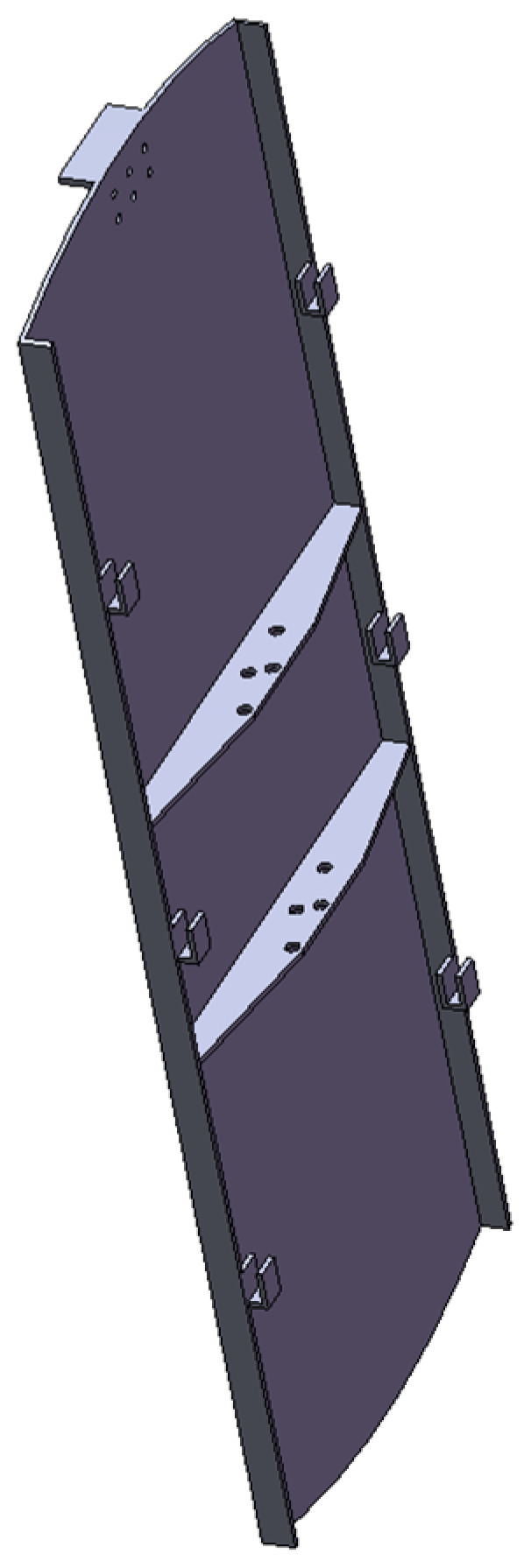
Plugging plate model.

The plate plugging robot used in this work is a rigid model, and in order to successfully enter the manhole, it is divided into three parts, with the robot performing three movements in order to feed it into the evaporator. Because the path planning process is similar for the three movements, only the middle plate with the largest size, measuring roughly 1054 mm × 354 mm and weighing around 10 kg, is selected for the path planning study. The plugging plate model is shown in Figure 3.

**Figure 4:**
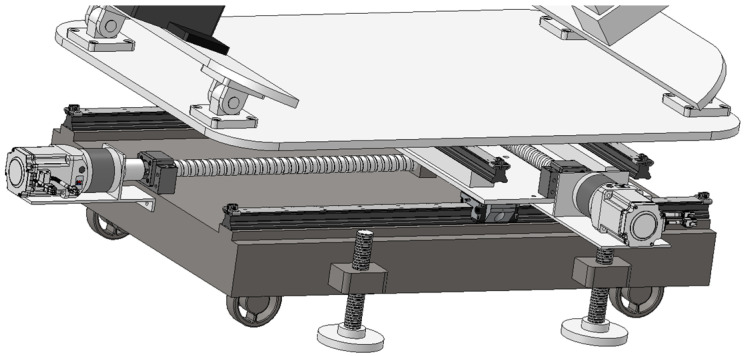
Screw module.

The aim of the steam generator plugging robot is to realize fully automated operation. The plugging robot is placed outside the manhole and installs the plugging plate into the plugging hole by means of a robotic arm. Due to the fact that the center point of the manhole is roughly 1.5 m from the ground, which is larger than the working radius of the general collaborative robotic arm, it is necessary to design a lifting device to send the robotic arm into the manhole. The plugging plate installation robot designed in this study consists of a robotic arm, two screw modules, two electric cylinders, and a trolley, which serves as the chassis of the robot and can be moved back and forth to facilitate the deployment of the robot to a specified location. A moving platform controlled by two screw modules is mounted on top of the trolley, controlling the robot arm to allow it to move back and forth, providing two degrees of freedom of movement for the robot, as shown in Figure 4. The upper end of the platform is connected to a feed cylinder and an angle adjustment cylinder, which are responsible for the lifting motion and telescoping motion of the front-end robotic arm, providing one degree of freedom of movement and one degree of freedom of rotation. The robotic arm is mounted at the end of the feed cylinder, providing six rotational degrees of freedom. The physical version and model of the plate plugging robot are shown in Figure 1b and Figure 2b. The robot as a whole has 10 degrees of freedom moving in conjunction with one another to accomplish the task of installing the plugging plate.

**Figure 5:**
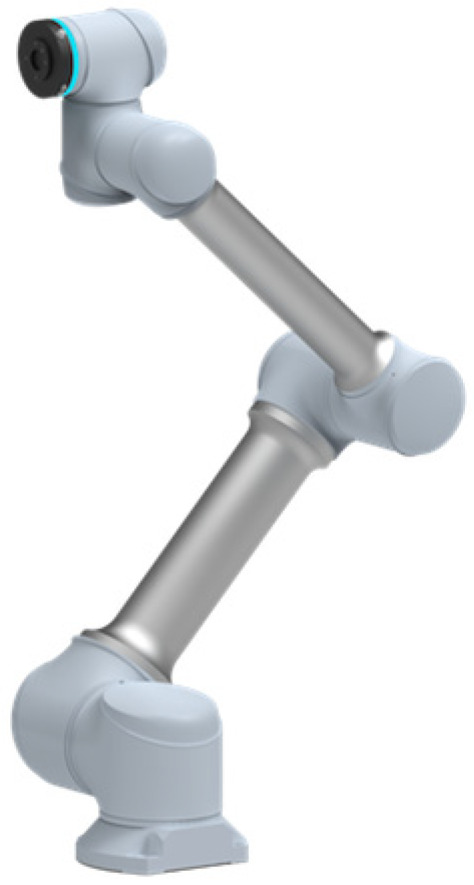
Xinsong GCR14-1400 six-degree-of-freedom collaborative robot.

The robotic arm is a Xinsong GCR14-1400 (SIASUN, Shenyang, China) six-degree-of-freedom collaborative robot, as shown in Figure 5. It can reach any position in the workspace. The robot arm has six rotating joints, and the angle range of each rotating joint is [−175°, 175°], including an automatic locking function, which has a vital role in its use in nuclear environments. The robot body weighs 14 kg and has a deadweight of 50 kg. Excluding the cylinder space directly above and below the base, the maximum working range of the robot is a radius of 1400 mm. The feed cylinder is a Normanson brand DDA95-5-X800 (NOUMASE, Kunshan, China) electric cylinder with a stroke of 800 mm, a thrust force of 140 kg, and a speed of up to 250 mm/s. The angle adjustment cylinder is a DDA95-5-X800 electric cylinder with a stroke of 800 mm, a thrust force of 140 kg, and a speed of up to 250 mm/s. The robot is also equipped with another angle adjustment cylinder, which is a DDA65-5-X350 (NOUMASE, Kunshan, China) with a stroke of 350 mm, a thrust of 140 kg, and a speed of 250 mm/s or lower. The screw module is driven by a Panasonic servo motor MHMF082L1U2 (Panasonic, Osaka, Japan).

### 2.2. Forward and Reverse Kinematic Analysis

**Figure 6:**
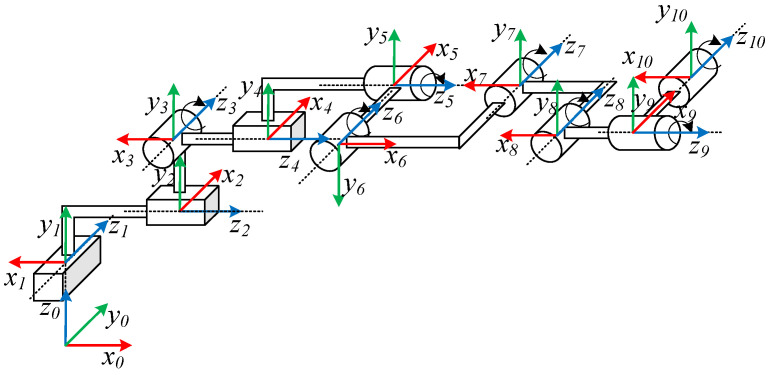
Plugging robot coordinate relationship diagram.

The plate plugging robot described in this study has seven rotational degrees of freedom and three translational degrees of freedom, and the relationship diagram of each coordinate system is shown in Figure 6. The D-H parametric method [29] is applied to establish its linkage coordinate system, and the connecting rod D-H parameters were obtained, as shown in Table 1.

In Table 1, a_i_ denotes the projected distance from z_i−1_ to the z_i_ axis along the direction of the x_i_ axis; α_i_ denotes the angle of rotation around the x_i_ axis that turns the z_i−1_ axis in the direction of the z_i_ axis; d_i_ denotes the projected distance from the x_i−1_ axis to the x_i_ axis along the direction of the z_i−1_ axis; and θ_i_ denotes the angle of rotation around the z_i−1_ axis that turns the x_i−1_ axis in the direction of the x_i_ axis. Mapping the vector described in the coordinate system {i} to the coordinate system {i − 1}, the transformation matrix can be written as

(1) Tii−1=cθi−sθicαisθisαiaicθisθicθicαi−cθisαiaisθi0sαicαidi0001

where sθ_i_, cθ_i_, sα_i_, and cα_i_ denote sinθ_i_, cosθ_i_, sinα_i_, and cosα_i_, respectively. The chi-square transformation matrix for each linkage can be obtained by bringing the parameters into the equation. Then, the position of the end effector with respect to the base scale is expressed as

(2) T100=T10T21T32T43T54T65T76T87T98T109=r11r12r13pxr21r22r23pyr31r32r33pz0001

where the matrix $X=\left[\begin{array}{ccc} r_{11} & r_{12} & r_{13}\\ r_{21} & r_{22} & r_{23}\\ r_{31} & r_{32} & r_{33} \end{array}\right]$ denotes the robot end pose and $\left[\begin{array}{ccc} p_{x} & p_{y} & p_{z} \end{array}\right]$ denotes the position vector of the origin of the robot end-effector coordinate system relative to the base coordinate system. By substituting the D-H parameters of the overhaul robot, the 3D coordinate points corresponding to the robot end effector can be found.

The inverse kinematics of the robot transform Cartesian space into joint space by understanding the desired position and attitude of the end effector relative to the base coordinate system and finding the joint angles that satisfy the desired position and attitude [30].

Using the inverseKinematics function, the user needs to first define a robot model (usually a rigidBodyTree object) and then call the function to compute the specific angles of each joint by combining the end effector’s target position and the initial guess value. Its iterative process automatically adapts to the multi-joint coupling characteristics of the robotic arm and satisfies the physical constraints of the arm while optimizing the objective. For redundant robotic arms in particular, inverseKinematics can allocate weights through a cost function to prioritize the optimal solution among multiple possible solutions, thus balancing solution efficiency and accuracy. This function is remarkably flexible and practical, especially for complex tasks such as path planning and obstacle avoidance operations. For example, in path planning, the joint configurations of each point can be calculated step by step according to the target points in the planned path in order to realize accurate matching between the path and the action. In obstacle avoidance operation, inverseKinematics combined with obstacle avoidance algorithms can ensure that the end effector avoids obstacles in complex environments while maintaining the accuracy of the operation. In this study, inverseKinematics functions are used for the inverse kinematics solution of the plate plugging robot.

## 3. Simulation Environment Construction

**Figure 7:**
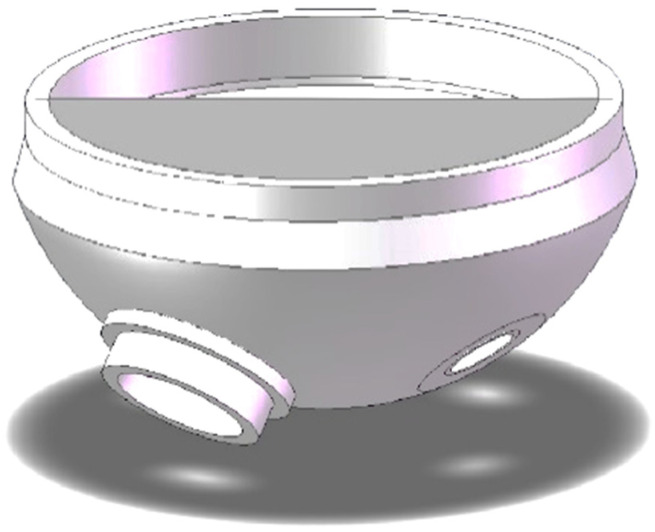
Simplified model of a steam generator.

**Figure 8:**
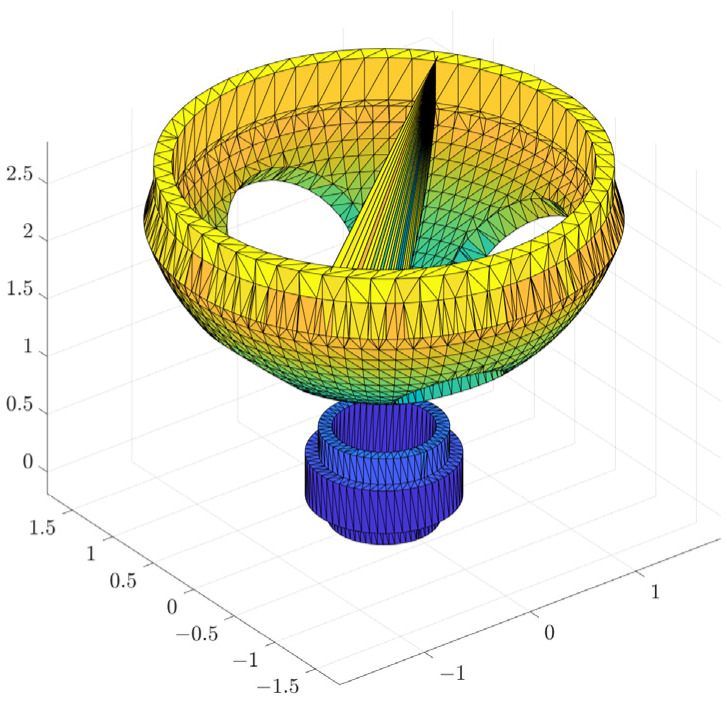
Steam generator stl format.

The simplified model of the steam generator is shown in Figure 7, where the manholes, plug holes, and bulkheads are modeled separately while the structure is simplified. The simplified model is exported as an stl file as input to the approximate convex decomposition algorithm, as shown in Figure 8.

Commonly used methods for fitting obstacle models include convex decomposition, bounding box, and mesh voxelization. Due to the complexity of the steam generator structure, it is difficult for the bounding box method to accurately fit the shape of the complex model, which may lead to a lack of accuracy; in contrast, the mesh voxelization method is computationally inefficient when high accuracy is required. The convex decomposition method can maintain the model’s shape more accurately by decomposing the model into multiple convex polyhedra, which is especially suitable for irregular structures and has high efficiency in collision detection. Therefore, the convex decomposition method was chosen to fit the obstacles in this study. Volumetric Hierarchical Approximate Convex Decomposition (V-HACD) is a convex decomposition algorithm for 3D models [31], the basis of which is to decompose a complex 3D model into a set of small pieces of approximate convex shapes in order to improve the efficiency of collision detection and physical simulation. The algorithm can effectively decompose a complex 3D model into a set of convex polygons or convex polyhedra while maintaining the overall shape and surface details of the model as far as possible. The algorithm first needs to voxelize the model to calculate the occupancy mesh. Once voxelization is computed, the algorithm continues to compute the convex components by recursively splitting the volume into two parts. First, it is centered and aligned in the coordinate system according to its principal axes. Then, one of the three axis-aligned planes is chosen as the splitting plane to divide the volume into two distinct parts. This process is repeated several times until we reach the maximum number or accuracy of convex packets required. The concavity η(C) of a set of components C is calculated as

(3) $$\eta (C)=\underset{k=1,\cdots K}{\max}d(C_{k},CH(C_{k}))$$

where d(X, Y) is the difference between volume X and volume Y; CH(X) is the convex envelope of X; C_k_ is the kth element in the set C; the partition plane is used as the partition plane by choosing the axis-aligned plane that minimizes the energy E(V, p); V is the volume that we want to partition; and p is the partition plane. The energy is defined as

(4) $$E(V,p)=E_{con}(V,p)+\alpha E_{bal}(V,p)+\beta E_{sym}(V,p)$$

where E_con_ is a component connectivity term that measures the sum of the normalized concavities between the two sides of the volume; E_bal_ is a balanced component term that measures the difference between the two sides; and E_sym_ is a symmetric component term that penalizes planes that are orthogonal to the potential rotation axis. The parameters α and β are the weights of the last two terms. The formulas for each component are given below:

(5) $$E_{con}(V,p)=\frac{d(V_{left},CH(V_{left}))+d(V_{right},CH(V_{right}))}{V_{0}}$$

(6) $$E_{bal}(V,p)=\frac{abs(\left|V_{left}\right|-\left|V_{right}\right|)}{V_{0}}$$

(7) $$E_{sym}(V,p)=w(\delta \cdot p)$$

where V_left_ and V_right_ are the left and right subparts after partitioning the plane p; $V_{0}=CH(S)$ is the volume of the convex packet of the polyhedral surface S; abs(x) is an absolute value of x; [V] denotes the volume of the original set V; δ is a potential axis of rotation; and w is a weighting factor describing how close δ is to the actual axis of rotation. In all of our experiments, we used the default value of α = β = 0.05.

**Figure 9:**
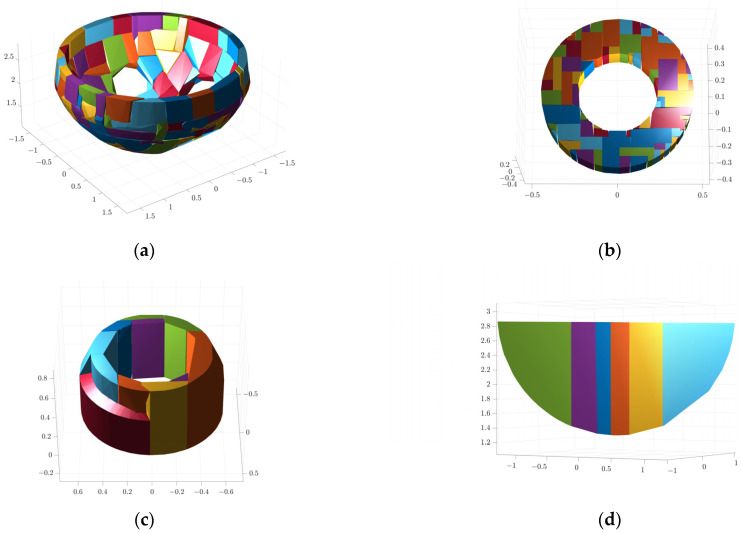
Results of V-HACD fitting: (**a**) main body; (**b**) manholes; (**c**) plug hole; and (**d**) bulkhead.

**Figure 10:**
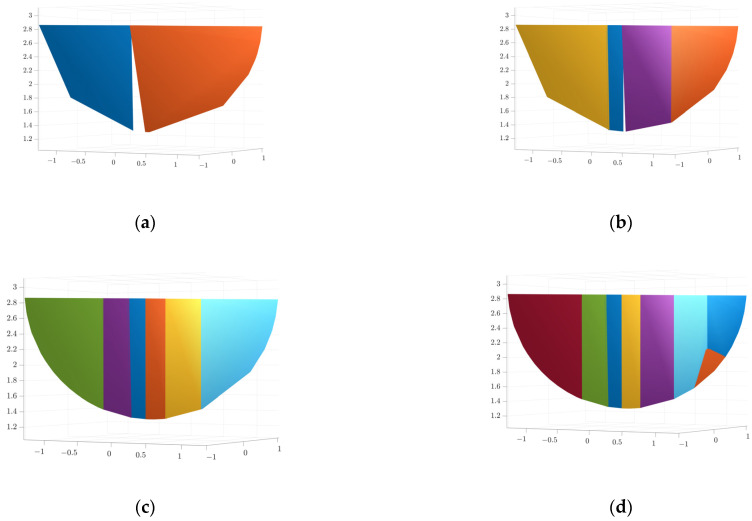
Results of fitting the bulkhead using different quantities of convex packets: (**a**) 2; (**b**) 4; (**c**) 6; and (**d**) 8.

**Figure 11:**
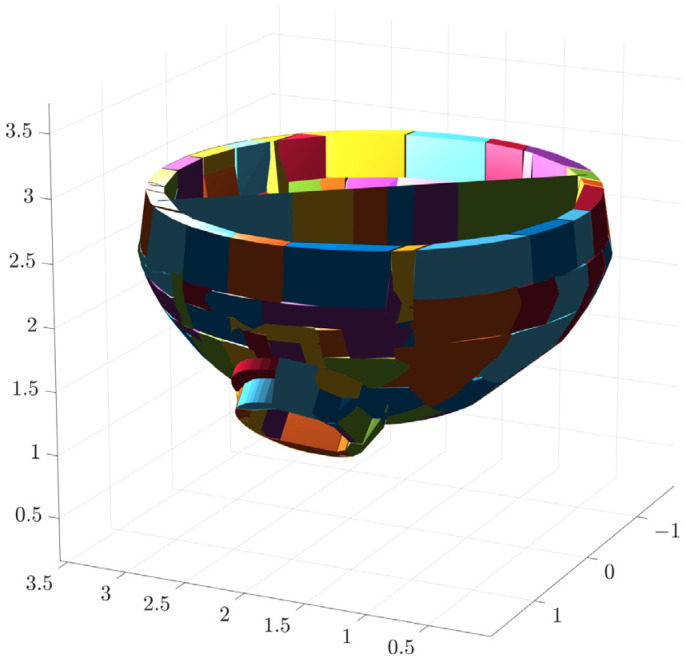
Overall fitting results of steam generator.

The results of convex packet fitting of the steam generator components using V-HACD are shown in Figure 9. Different quantities of bumps were used to fit the water chamber, manholes, plug holes, and bulkheads to pick the most appropriate quantity of bumps. As can be seen in Figure 10, when fitting the bulkhead, different quantities of bumps have a greater impact on the results after fitting; using two or four bumps will lead to gaps in the fitting results, affecting the accuracy of the simulation. In contrast, using eight bumps will lead to redundancy in the fitting results, reducing the efficiency of the simulation. In light of these findings, the quantity of bumps selected for fitting was six. Considering the complexity of the fitted results and the impact on the subsequent simulation process, the quantities of fitted bumps for the main body, manhole, plug hole, and partition were 96, 64, 16, and 6, respectively. The fitted results were subjected to a series of coordinate transformations so that they could be assembled together to form a complete steam generator, as shown in Figure 11.

**Figure 12:**
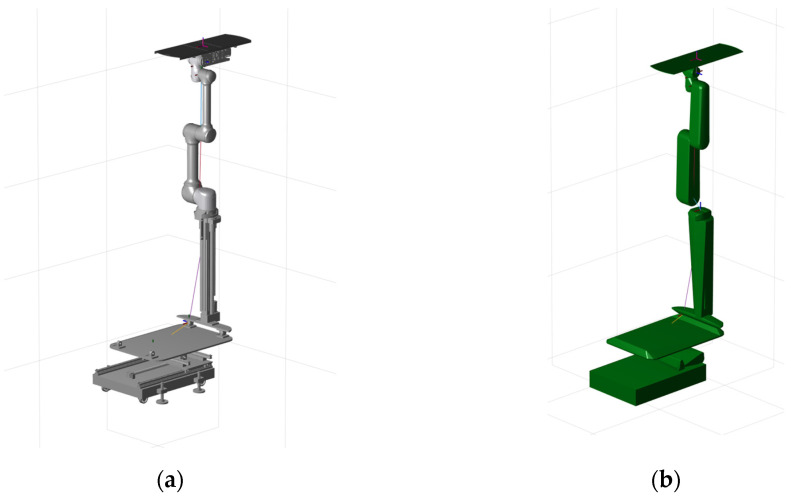
Model fitting for the plate plugging robot: (**a**) original model and (**b**) model after convex packet fitting.

**Figure 13:**
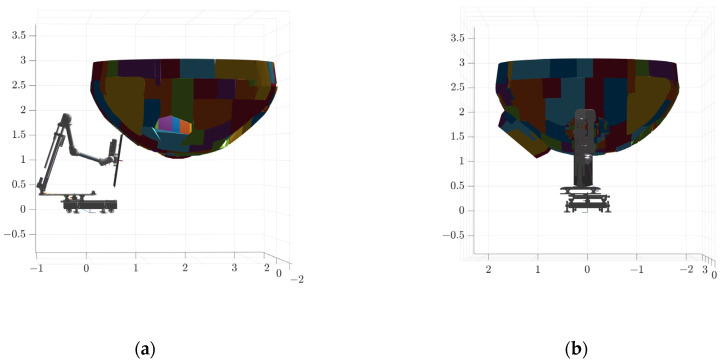
Initial attitude of the plate plugging robot: (**a**) side view and (**b**) front view.

Due to the fact that the structure of the plate plugging robot is also relatively complex, the collision model of its components was also simplified using V-HACD, and the quantity of bumps was set to two, with the results shown in Figure 12. The plate plugging robot model was imported into the steam generator and its initial position was set, as shown in Figure 13. The values of the joints of the plate plugging robot are [0, 0.1, −0.50, 0, π, 2.27, 1.83, 0, 0, π/2], and the coordinates of the center point of its base are (0, 0, 0); the coordinates of the center point of the water chamber of the steam generator and the partition plate are (1.786, 0, 0); the coordinates of the center point of the manhole are (0.59922, 0, 1.54726), with the hole orientation set to (0, −140°, 0); and the coordinates of the center point of the plug hole are (1.75172, 0.98158, 1.79109), and the orientation of the hole is (−140°, 0, −90°).

## 4. Path Planning Simulation

### 4.1. Rapidly Exploring Random Tree Algorithm

In high-radiation or hazardous environments, real-time path planning is prioritized over optimality; in comparison, in repetitive robotics tasks, only one feasible path needs to be found each time for path planning without strict optimization. The core idea of the RRT algorithm is to build a random tree and expand it randomly from the starting point until the branches grow to the target point. The RRT algorithm is able to quickly find an efficient path from the starting point to the goal in complex environments and can avoid the local minimum problem. For multi-degree-of-freedom blocking robots in particular, the RRT algorithm is better adapted to the requirements of path planning in high-dimensional spaces than traditional A* algorithms, for example, and compared with current state-of-the-art algorithms, the RRT algorithm quickly explores high-dimensional complex environments and generates feasible paths at a lower computational cost, which is well suited for the application scenarios in this study.

### 4.2. Phased Path Planning

After analyzing the actual workflow, it can be seen that the entire process has increased in complexity. If overall path planning is carried out, the calculation process is complex, and it is difficult to obtain effective results. Therefore, the analysis process is divided into three separate stages. The path of each stage is calculated and then the paths calculated in the three stages are added together to complete path planning for the installation process of the plugging plate.

**Figure 14:**
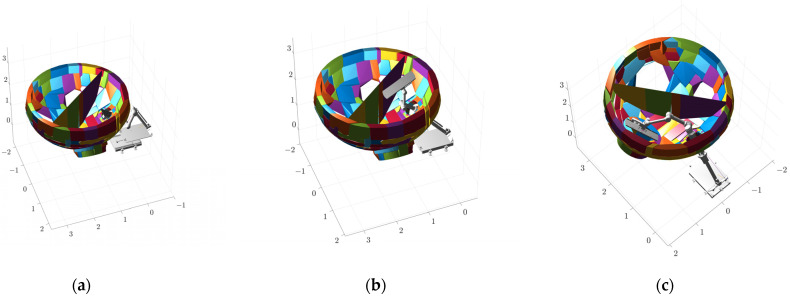
Positions reached by each stage of the plate plugging robot: (**a**) Phase I; (**b**) Phase II; and (**c**) Phase III.

The first stage involves the plate plugging robot clamping the plugging plate and feeding it into the manhole from the initial height before moving to the position shown in Figure 13a. The values of each joint are [0, 0.1, −0.70, 0, π, 0.82, 0.87, 0, 0, π/2], of which the unit of the first, second, and fourth joints is m, and the unit of the other joints is rad. The end effector has the coordinates under the Cartesian coordinate system as (0.9323, −0.0094, 2.1496) and the target orientation is (−180°, 53°, 0). Firstly, given the initial and end position, the values of each joint of the initial position are [0, 0.1, −0.50, 0, π, 2.27, 1.83, 0, 0, π/2], the coordinates of the end effector are (0.4810, −0.0094, 1.5443), and the target orientation is (−180°, 36.5°, 0). Interpolation operations are used to optimize the results of path planning and generate smoother and more continuous paths for subsequent path tracking and robot control applications. In this experiment, we set the interpolation factor to five, indicating that five additional interpolation points are inserted between every two path points. The trajectory of the first stage is shown in Figure 14a. The green line in the figure indicates the trajectory of the end effector, and each circle corresponds to a bit position of the robot. Obstacles and some parts of the robot are hidden for ease of observation.

**Figure 15:**
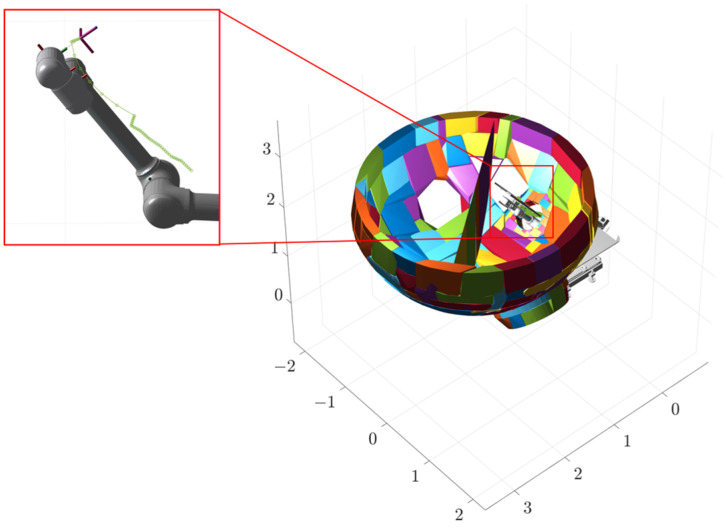
Phase I trajectory.

**Figure 16:**
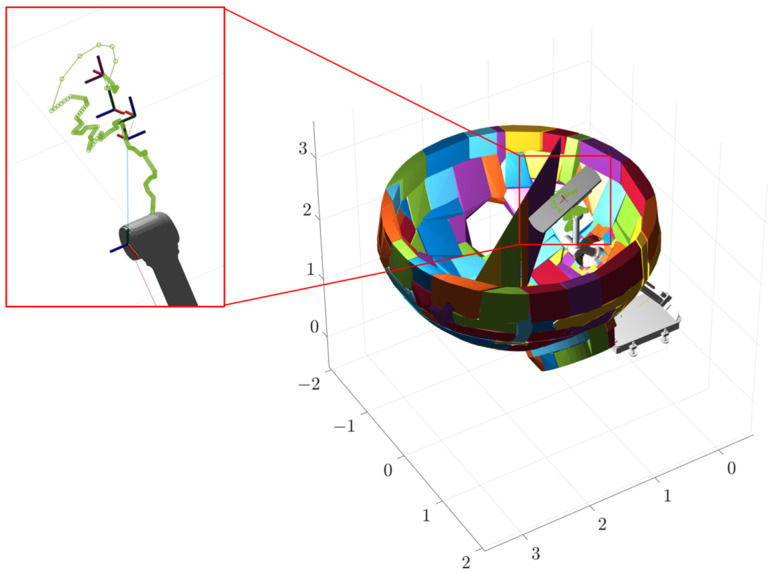
Phase II trajectory.

**Figure 17:**
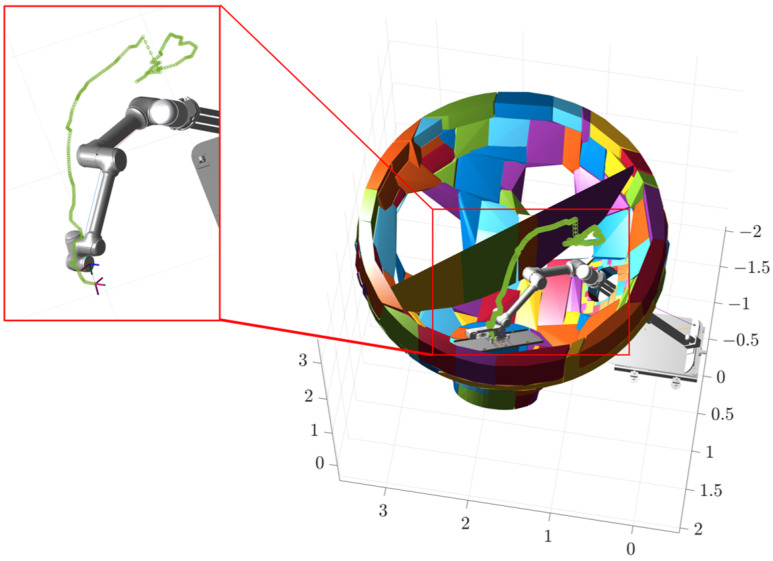
Phase III trajectory.

The second stage takes place inside the steam generator, where the robotic arm performs a series of movements to flip the plugging plate and adjust it to the optimal attitude, as shown in Figure 14b, at which time the values of the joints are [0.5, 0.315, −0.70, 0.10, 0, −0.17, −0.87, −0.52, 0, 0], the coordinates of the end effector are (1.1636, 0.0828, 2.7832), and the target orientation is (0, 30°, 0). The third stage involves covering the plugging plate at the specified position of the plug opening, as shown in Figure 14c, at which time the values of the joints are [0.5, 0.2, −0.70, 0.70, 0.96, −0.61, π/4, −π/4, 0, 0], the end-effector coordinates are (1.7812, 0.9888, 1.8415), and the target orientation is (140°, 0, −90°). The second and third segments of the path planning simulation process are identical to the first segment, both using interpolation operations with an interpolation factor of five. Their working trajectories are shown in Figure 15, Figure 16 and Figure 17.

**Figure 18:**
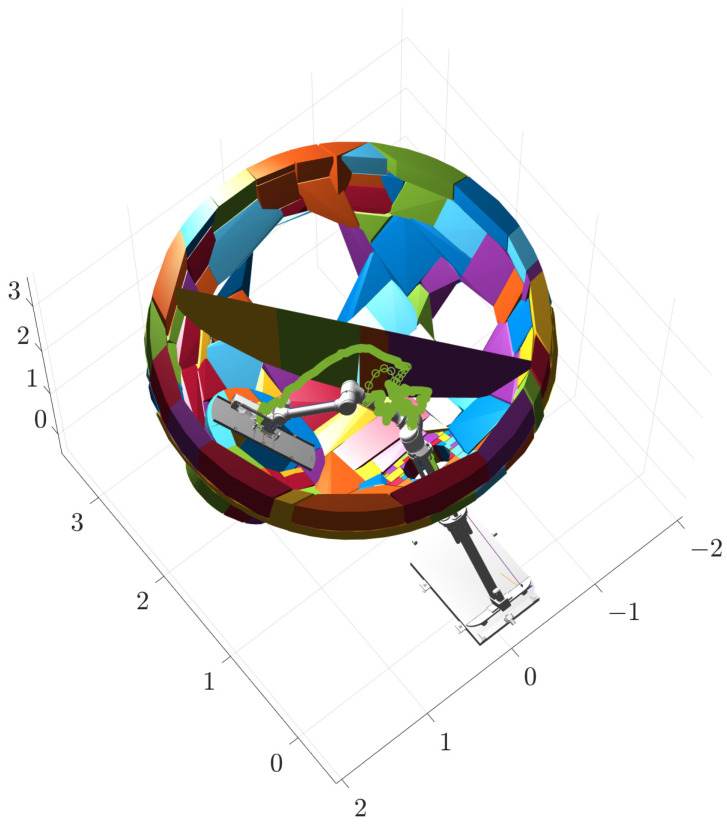
Overall phase trajectory.

**Figure 19:**
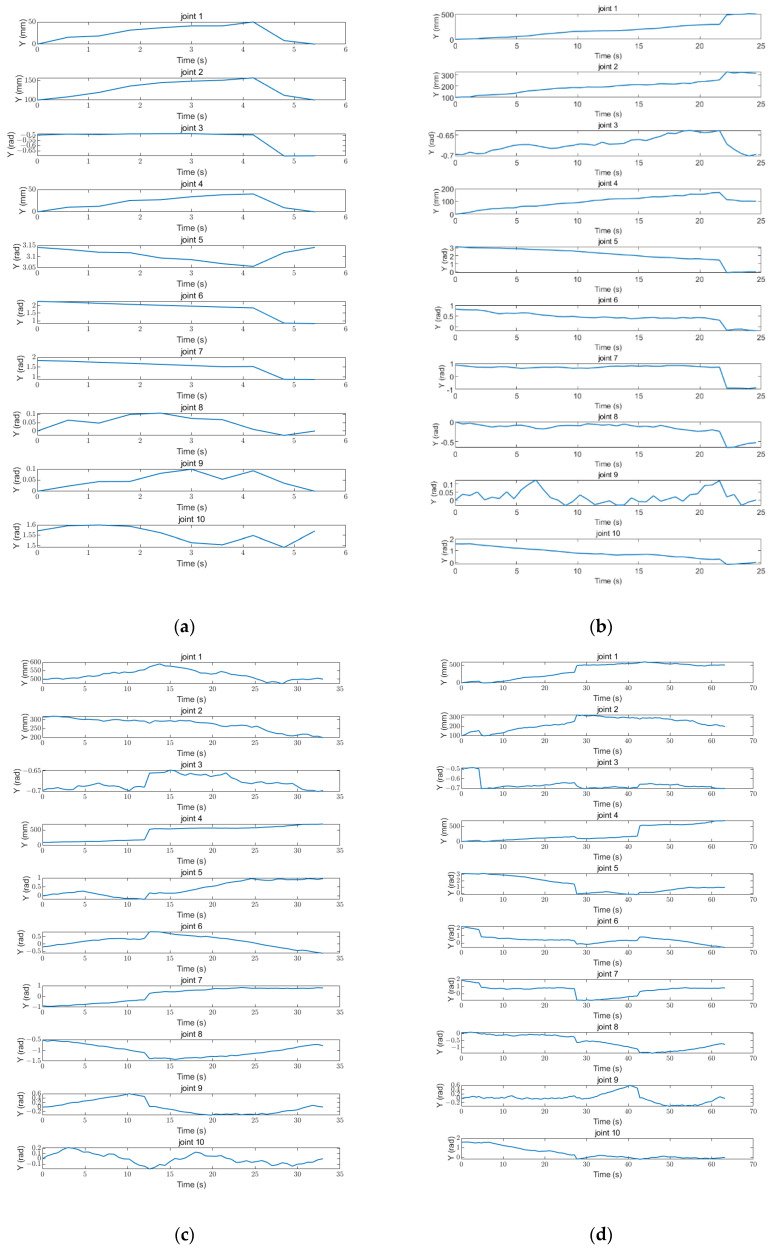
Variation in parameters in each joint: (**a**) Phase I; (**b**) Phase II; (**c**) Phase III; and (**d**) overall.

The three stages of the path are integrated to obtain the complete path, as shown in Figure 18. The variation curves of the joint parameters for each stage and the entire process are shown in Figure 19.

## 5. Discussion of Experimental Results

This study focuses on obstacle avoidance path planning simulation using the Robotics System Toolbox in MATLAB R2024a [32]. The Robotics System Toolbox is a development tool in MATLAB dedicated to the modeling, simulation, and control of robotic systems. It provides a rich set of functions and algorithms to handle tasks in robot kinematics, dynamics, path planning, collision detection, control design, etc. The simulation results show that the robot’s path is relatively smooth in the first phase and the changes in the parameters of the joints are also relatively low. The path characteristics indicate that the robot is able to maintain better stability and coordination in this stage when performing simpler tasks or moving in a relatively simple environment. The smooth path not only helps to reduce energy consumption but also reduces the wear and tear on each joint and prolongs the life of the robot. By analyzing the parameters of this phase, we can see that the robot moves more efficiently and completes tasks faster during this time.

In the second stage, the path starts to exhibit zigzagging; in particular, the parameter change curve of joint 9 is evident. This phenomenon indicates that the robot may have encountered a more complex task or working environment in this phase. The zigzagging nature of the path reflects the fact that the robot needs to frequently adjust its attitude and position to adapt to changes in the environment or to fulfill specific tasks. The parameter variation in joint 9 is particularly significant, suggesting that this joint plays a key role in task execution. In order to improve the path smoothness in the second stage, it will be necessary to deeply analyze the motion characteristics of joint 9 and optimize its control strategy.

The simulation results of the third phase show the same zigzag paths; in particular, the parameter variation curves of joint 3 and joint 10 are particularly significant. This phase may involve more complex task requirements or more complicated environmental conditions. The dramatic changes in joints 3 and 10 may be closely related to postural adjustment and precise manipulation of the robot. By analyzing the motion data of these two joints in detail, we can further optimize the motion control algorithms of the robot to ensure that it operates more smoothly and efficiently when facing complex tasks.

**Figure 20:**
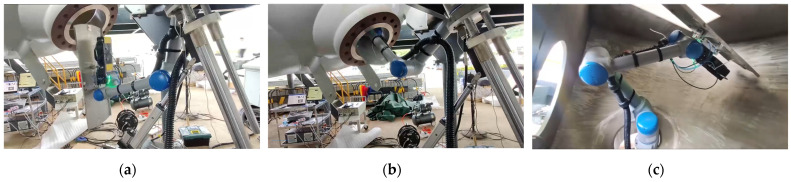
Robot teaching process: (**a**) initial pose; (**b**) access to manhole; and (**c**) posture.

In Figure 20, the teaching process is shown, and it can be seen that the chosen teaching method often requires manual step-by-step guidance of the robotic arm to move into various postures and positions. This method requires precise manual debugging for each action, which is time-consuming and more prone to errors in judgment. Especially when in close proximity to obstacles, the taught path cannot guarantee the best safety distance, increasing the risk of damage to the equipment. Our method automatically generates paths through the path planning algorithm without manual step-by-step adjustments, which effectively shortens the operation time. Simultaneously, it can accurately avoid obstacles to ensure safe operation, especially in complex environments to enhance safety. The teaching method is suitable for fixed environments; however, once the environment or the equipment changes, retraining and adaptation are necessary. Our method can adapt to different working environments and equipment configurations through parameter adjustment, which is more flexible and avoids repetitive manual guidance. Manual methods rely on the operator’s experience and skills, generating significant uncertainty and risk; in comparison, demonstrative methods require the operator to perform demonstrative operations in a high-radiation environment, representing a potential safety hazard. Through automated path planning, the plate plugging robot is able to complete the task at hand without any intervention, which greatly reduces the risk of the operator being exposed to a high-radiation environment and improves work safety and efficiency. Although the simulation results show that the path is not smooth enough, we successfully obtained detailed data on the entire working path of the plate plugging robot. These data have direct application value for path planning and robot control in real environments. By analyzing and applying these data, we have not only solved the issue of path planning by plate plugging robots during their workflow but also provided a valuable reference for subsequent optimization. Our future research efforts will focus on detailed robotics experiments to verify the practicality and performance of the methods described in this paper.

## 6. Conclusions

In this study, to address the requirements for the disassembly of steam generator plugging plates, we designed the body structure of a plate plugging robot with ten degrees of freedom based on the linkage D-H parametric coordinate system. We also established its forward and inverse kinematics equations and ultimately solved them. The robot’s structure and experimental environment were modeled using modeling software and then imported into simulation software, where we carried out the simulation of trajectory planning. The results show that the robot operates normally within the set range, which provides a foundational argument for the plate plugging robot program and provides an important theoretical basis for the use of robots to replace traditional manual operations in order to improve the safety and efficiency of the entire process of plate plugging and disassembly. Although the current algorithm still has room for improvement in terms of path smoothness and motion efficiency in complex environments, future research efforts are expected to significantly improve the performance of the plate plugging robot by optimizing the algorithm and improving the joint control strategy. Through continuous research and practice, smoother and more efficient robot path planning can be achieved, providing more reliable technical guidance for plate plugging robots in practical applications.

## Figures and Tables

**Table 1:** D-H parameters.

Joint	θ_i_/rad	d_i_/m	a_i_/m	α_i_/rad
1	0	d_1_	0.2877	π/2
2	0	d_2_	0.6392	0
3	θ_3_	0.2900	0.2636	π/2
4	0	d_4_	0.2607	−π/2
5	θ_5_	0.0820	0	π/2
6	θ_6_	0.0985	0.1087	0
7	θ_7_	0.6492	0.0033	π/2
8	θ_8_	0.6125	0.0595	−π/2
9	θ_9_	0.0780	0.0780	π/2
10	θ_10_	0.0595	0	0

## Data Availability

The data presented in this study are available from the corresponding author upon request.
